# Lessons from a Severe Case of Fulminant Guillain–Barré Syndrome: A Case Report

**DOI:** 10.3390/reports9020138

**Published:** 2026-04-29

**Authors:** Jacob Allen Saunders, Sadiq Shakir Patel, Thomas Chandy Varkey, Sara Shaikh, Anthony Conforti, Ganesh Murthy

**Affiliations:** 1 Department of Neurology, The University of Arizona College of Medicine—Phoenix, Phoenix, AZ 85004, USA; 2 Department of Internal Medicine, The University of Arizona College of Medicine—Phoenix, Phoenix, AZ 85004, USA

**Keywords:** GBS, severe disease, AIDP, autoimmune neurology, case report

## Abstract

**Background and Clinical Significance**: Guillain–Barré syndrome (GBS) can rarely progress to fulminant paralysis with loss of brainstem reflexes, mimicking coma or brain death despite preserved cortical function. **Case Presentation**: A 38-year-old man developed rapidly progressive weakness following a diarrheal illness, culminating in quadriplegia, areflexia, respiratory failure, and complete loss of brainstem reflexes within 72 h. Neuroimaging was unrevealing. EEG demonstrated preserved cerebral activity with an alpha coma pattern. Despite initial intravenous immunoglobulin therapy, neurological deterioration continued, prompting escalation to plasma exchange. Gradual recovery of brainstem reflexes and motor function ensued, followed by substantial functional improvement over nine months. This case highlights the diagnostic and prognostic challenges of fulminant GBS at the interface of peripheral and brainstem dysfunction. Neurophysiologic assessment and disciplined exclusion of central etiologies are essential. Timely immunotherapy and supportive care can lead to meaningful recovery even in extreme presentations. **Conclusions**: Fulminant GBS should be recognized as a potentially reversible cause of apparent coma, underscoring the importance of early diagnosis and aggressive treatment.

## 1. Introduction and Clinical Significance

Guillain–Barré syndrome (GBS) is an acute, immune-mediated polyradiculoneuropathy in which the defining clinical problem is rapid-onset peripheral nerve and nerve root dysfunction. Core clinical features include progressive weakness with hyporeflexia or areflexia often accompanied by sensory symptoms, pain, cranial nerve involvement, respiratory insufficiency, and autonomic dysfunction. The severity of disease and complication burden can vary widely among patient presentations. Meaningful variation in the tempo and distribution creates diagnostic challenges [1,2].

GBS is an umbrella diagnosis that contains several recognizable phenotypes [1,2,3,4]. Presentations can range from regional or variant forms to generalized weakness. Notable variants include Miller Fisher syndrome (MFS) and the pharyngeal–cervical–brachial (PCB) variant. MFS is defined by ophthalmoplegia, ataxia, and areflexia while PCB primarily affects bulbar and upper limb musculature. Overlap syndromes exist between MFS and PCB variants and generalized GBS [1,2,3,4]. GBS phenotypes also vary in the electrophysiologic pattern of injury. Classic electrophysiologic categories are acute inflammatory demyelinating polyradiculoneuropathy (AIDP) and the axonal forms: acute motor axonal neuropathy (AMAN) and acute motor–sensory axonal neuropathy (AMSAN) [2,3,4].

Regardless of the pattern of injury and anatomic distribution, severity is best defined by functional trajectory and physiologic threat rather than by subtype label alone. Any phenotype can deteriorate rapidly and early complications especially respiratory failure and dysautonomia drive morbidity and clinical decision-making in the acute phase [1,2,4]. At the most severe end, fulminant GBS can abolish voluntary movement and peripheral reflexes and can also extinguish brainstem reflexes. This creates a clinical picture resembling coma or brain death despite preserved cortical activity. The literature describes cases in which brainstem reflexes were absent yet EEG showed remaining cerebral electrical activity and recovery occurred with supportive care and immunotherapy [5,6].

This report describes a case of GBS with rapid progression to fulminant GBS in a 38-year-old male patient with transient loss of all brainstem reflexes, preserved cerebral function on EEG, and a remarkable long-term neurological recovery.

## 2. Case Presentation

A 38-year-old man with a history of Poland syndrome, remote left Bell’s palsy, Bicuspid aortic valve, hypoplastic posterior mitral leaflet with associated lengthening anterior mitral leaflet, and ascending aortic aneurysm presented to an outside hospital with two days of progressive weakness in his hands and feet. Symptoms began approximately one week after resolution of a self-limited flu-like illness associated with diarrhea (Table 1). There was no reported sensory level, bowel or bladder incontinence, or recent toxin exposure.

Initial workup at an outside facility revealed a small acute infarct in the inferior left cerebellum and chronic left frontal encephalomalacia on magnetic resonance imaging (MRI) and computerized tomography (CT). Cervical spine MRI was unremarkable. A CT angiogram of the chest demonstrated a nodular infiltrate in the right lower lung and aortic dilation. Due to concern for a rapidly progressive neuromuscular process, the patient was intubated for airway protection and transferred to the treating institution with suspected GBS.

On arrival, the patient was evaluated under light sedation and was able to open his eyes to command. Neurologic examination demonstrated complete paralysis of the bilateral hands as well as plantarflexion and dorsiflexion. Minimal contractions were noted with hip flexion and extension. Elbow flexion and extension were antigravity and deltoid strength was preserved. Sensation was globally diminished and deep tendon reflexes were markedly reduced throughout. The Glasgow coma scale was 10 on arrival (4 for eyes, 6 for motor, T for verbal). A lumbar puncture performed on arrival was traumatic and nondiagnostic. CT of the head was obtained to exclude subarachnoid hemorrhage and was negative. Blood cultures were obtained at this time. The patient was started on intravenous immunoglobulins (IVIGs) due to a high clinical suspicion of GBS. Initial dose was 0.4 g per kilograms per day (g/kg/day). Differentials at the time included botulism and tick paralysis though no tick bite was identified and the ascending progression of weakness conflicted with the classic botulism presentation.

On hospital day 2, despite starting IVIG and given a total of 0.8 g per kilograms (0.4 g per kilograms per day), the patient progressed to flaccid quadriplegia rated as 0/5 strength in all muscle groups with complete areflexia. Within 48 h of arrival, the patient had lost all brainstem reflexes including pupillary light responses, corneal reflexes, oculocephalic responses, and gag reflex. Sedation had been minimized and no neuromuscular blocking agents were in use. The Glascow coma scale at that time was 3 (1 for eyes, 1 for verbal, and 1 for motor). A code stroke was activated due to the abrupt change in examination; repeat neuroimaging showed no new ischemic or hemorrhagic lesions. EEG demonstrated preserved cerebral electrical activity with a nonreactive alpha-frequency background consistent with an alpha coma pattern without epileptiform activity or evidence of cortical suppression. These findings supported preserved cortical function despite the profound brainstem and peripheral neurologic deficits. Given rapid progression and lack of response to IVIG, plasma exchange was initiated. The patient underwent 10 total sessions of plasma exchange with 3 L of plasma being removed and replaced with 3 L of 5% human albumin. Prior to each session he was given 50 mg of diphenhydramine to prevent allergic reactions and a fibrinogen level was obtained to determine if the patient needed to have human blood products to replace his fibrinogen levels. The machine utilized was a Spectra Optia^®^ (TerumoBCT 10811 W. Collins Avenue, Lakewood, CO 80215, USA) Apheresis System which is a continuous-flow centrifugation machine. The sum total of all plasma that was exchanged over the 10 sessions was 30 L of plasma.

**Figure 1:**
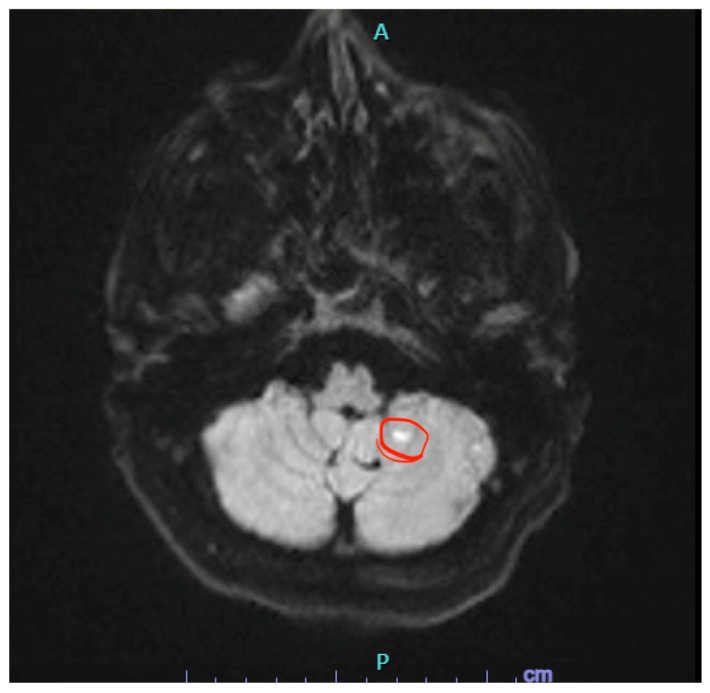
Diffusion-weighted imaging magnetic resonance image demonstrating the small stroke of the left cerebellum.

**Figure 2:**
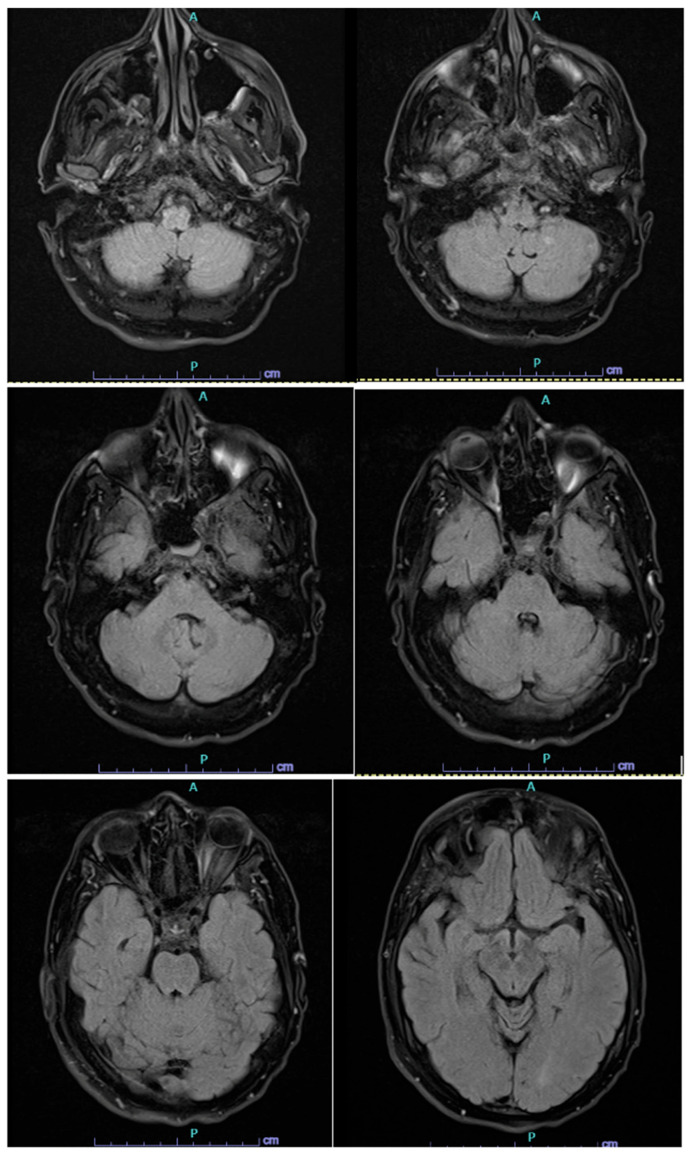

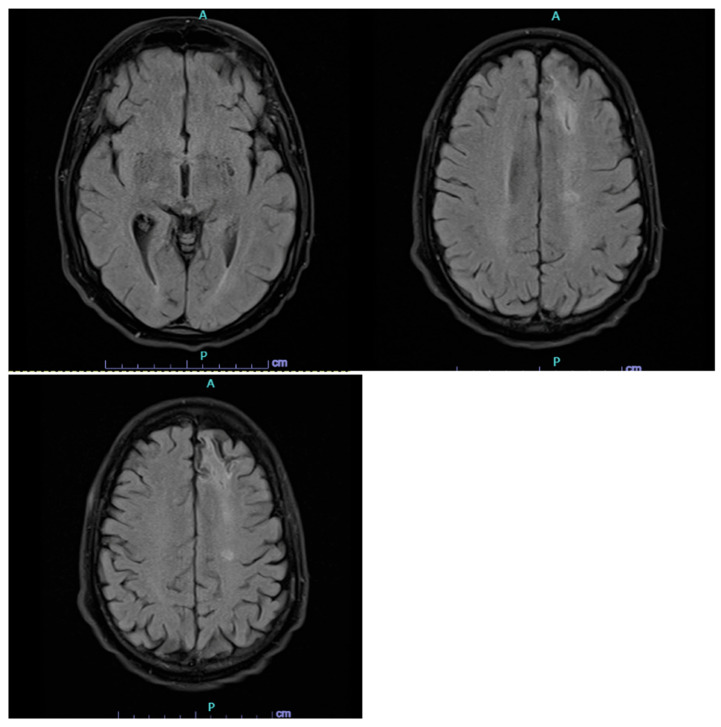
T2 fluid attenuated inversion recovery magnetic resonance images of the brain from most inferior to most superior with evidence of the new left-sided acute infarct and left-sided frontal lobe encephalomalacia. There is no evidence of encephalitis or inflammation on this image set.

A repeat lumbar puncture demonstrated mild pleocytosis and elevated protein. Cerebrospinal fluid studies were negative for West Nile virus, anti-GM1 antibodies, anti-GQ1b antibodies, the autoimmune encephalitis panel with ARUP laboratories, and MuSK antibodies. A Urine Porphobilinogen (PBG) and Plasma Porphyrin Scan were both run and negative. An echocardiogram was performed which demonstrated an ejection fraction of 37%. Further symptoms: unicuspid unicommissural aortic valve which appeared calcified and degenerated; severe aortic insufficiency and moderate aortic stenosis with mean gradient 21 mmhg; proximal ascending aorta severely dilated 5.1 cm; severe LVE with grade III diastolic dysfunction; diastolic mean gradient 8 mmhg; and diastolic reversal noted in the descending aorta consistent with aortic insufficiency and a small circumferential pericardial effusion. At this time the blood cultures grew out Strep Mutans which in the context of new onset aortic insufficiency and the pericardiac effusion seen on echocardiogram led to a diagnosis of subacute bacterial endocarditis, which per the ID physician was the likely cause of the GBS presentation and the stroke of the left inferior cerebellum. He was started on antibiotic treatment at that time with ceftriaxone. An MRI C/T/L Spine W+W/O was performed and was negative for abnormal enhancement, compression, or evidence of ischemic or demyelinating disease. There was no root thickening, cauda equina enhancement, or contrast enhancement of the nerve roots. A repeat MRI Brain W+W/O contrast was ordered and obtained and pertinent only for the known area of diffusion restriction in the left cerebellum (Figure 1 and Figure 2). Evaluations of the bitemporal region, the cranial nerves, the posterior fossa structures, and other areas were negative for signs of autoimmune encephalitis.

By hospital day 9, after four sessions of plasma exchange, the patient demonstrated early neurologic improvement with return of pupillary reflexes. Three days later, he regained the ability to activate sternocleidomastoid and trapezius muscles allowing head movement. A tracheostomy was placed on hospital day 15 for ongoing respiratory support. After completion of ten plasma exchange sessions, he exhibited limited extraocular movements without full scleral burial, jaw motion, tongue flickers with attempted movement, and weak head turning although diffuse flaccid paralysis persisted. On hospital day 22, a second abbreviated three-day IVIG course was initiated, as the initial IVIG course was only partially administered. This repeat dose was administered as 0.67 g/kg/day for three days. Following this, nursing staff reported subtle improvement in left elbow movement though this was not clearly appreciated on formal neurologic examination.

Three days after completing IVIG, the patient developed transient anisocoria and family-reported changes in responsiveness. CT of the head revealed a small right parietal subarachnoid hemorrhage without mass effect which was attributed to the bacterial endocarditis for which the patient was on antibiotic treatment and there were plans for surgical treatment depending on neurologic recovery. On evaluation by stroke neurology, anisocoria had resolved and follow-up imaging demonstrated no hemorrhage expansion. At that time, improved proximal strength was noted in all four extremities. Repeat MRI Brain without gadolinium contrast was pertinent only for the known subarachnoid hemorrhage in the parietal lobe region and based on the timeframe and location of the subarachnoid hemorrhage it was deemed that this had likely developed around the use of the plasmapheresis therapy and that this was not clinically relevant, so further diagnostics and therapeutics were not pursued. Given clinical stability and gradual neurologic improvement, the patient was discharged on hospital day 32 to a long-term acute care facility for continued ventilatory weaning and rehabilitation.

Over subsequent months, the patient demonstrated steady functional recovery with physical therapy. Six weeks after discharge, the patient was transferred back to the treating institution for elective aortic and mitral valve replacements with ascending aortic aneurysm repair related to a congenital heart disease. The surgery was uncomplicated and a percutaneous endoscopic gastrostomy tube was placed during this admission. The patient then returned to the long-term acute care facility for further rehabilitation.

At nine-month outpatient follow-up, the patient had regained full cranial nerve function and reflexes. He remained wheelchair-dependent but demonstrated substantial strength recovery in all extremities with proximal strength exceeding distal. Examination revealed rigidity and spasticity and he was referred to physical medicine and rehabilitation for evaluation. Four months later, after completing inpatient rehabilitation, the patient was ambulating independently. He reported residual urinary urgency, erectile dysfunction attributed to diminished sensation, and intermittent dysphagia with large food boluses but otherwise noted marked neurologic improvement. At his most recent follow-up, mild sensory loss was noted in the right L2–L3 dermatomes. MRI of the lumbar spine demonstrated degenerative disk disease at L4–L5, inconsistent with his symptoms. Neurosurgical evaluation did not recommend operative intervention. The patient continues to follow with physical medicine and rehabilitation for spasticity management and gastroenterology for dysphagia. All dates have been made very clear in Table 1.

## 3. Discussion

This case illustrates an extreme but reversible presentation of fulminant Guillain–Barré syndrome in which complete loss of brainstem reflexes closely mimicked coma or brain death despite preserved cortical function. In such presentations, disciplined confirmation of etiology and reversibility is essential before attributing the neurologic examination to primary central nervous system failure, particularly when neuroimaging does not explain bedside findings [5,6].

Conceptually, these presentations sit near the interface between peripheral neuromuscular failure and disorders with brainstem involvement demonstrating preserved cerebral activity and supporting a potentially reversible process, such as Bickerstaff brainstem encephalitis (BBE), which is closely related to the anti-GQ1b spectrum and is distinguished by impaired consciousness alongside ophthalmoplegia and ataxia [1,7]. In BBE, stereotyped EEG patterns including predominate N1 and N2 sleep patterns despite patient responsiveness have been reported that plausibly reflect dysfunction of the ascending reticular activating system, providing a framework for why consciousness and arousal can be affected even when cortical structure is intact [7]. The present case is mostly situated at the fulminant pole where peripheral and brainstem-level immune dysfunction can produce an examination that is disproportionate to structural imaging, making neurophysiology a critical adjunct to clinical reasoning [1,5,6,7]. While the clinical presentation and diagnostic studies suggested a condition within the realm of the anti-GQ1b spectrum, the exact cause was difficult to ascertain. Historically, it has been shown and discussed that electrophysiological studies, such as nerve conduction studies (NCS), are vital to differentiate the different types of GBS variants [8]. In the present case, a NCS was not performed during the acute hospitalization, thus limiting the ability to fully differentiate fulminant GBS from other possible variants. This represents a severe limitation of the diagnostic workup of this case.

Another abnormal finding within this case was the development of upper motor neuron (UMN) symptoms after the patient had been in rehabilitation for several months. There have been some reports previously of concomitant GBS and acute transverse myelitis, though these have been mostly reported in pediatric populations [9]. The proposed mechanisms for this finding include a common myelin epitope found in both the peripheral and central nervous systems, allowing for a common target for autoimmunity. Given that the patient’s spasticity was mostly located in the upper extremities and developed after his discharge, we unfortunately did not have a cervical spine MRI available to assess whether the patient’s symptoms may be provoked by a spinal lesion. It is possible that these findings may be related to the incidentally found cerebral infarcts during his initial hospitalization, though this seems less likely given the delayed presentation of the UMN symptoms. The last potential cause for the UMN signs of spasticity might be that of “pseudospasticity” as has been described in the literature before by Preston and Kelly [10], caused by peripherally generated continuous motor unit discharges; however, EMG was not performed in this patient and instead treatment with onabotulinumtoxin A was performed which has provided him significant benefit in the resolution of symptoms.

The evidence-based therapeutic foundation of GBS is timely immunomodulation plus meticulous supportive care. The EAN/PNS guideline recommends IVIG 0.4 g/kg daily for 5 days for patients within 2 weeks of onset who are unable to walk unaided with a good-practice window extending to 2–4 weeks, or plasma exchange totaling 12–15 L in 4–5 exchanges over 1–2 weeks for patients within 4 weeks of onset who are unable to walk unaided [1]. Treatment timing is repeatedly emphasized in modern practice. A 2024 study found that earlier IVIG initiation was associated with better outcomes and reported an “ideal time window” within the first 2 weeks with diminished therapeutic effect when IVIG was administered beyond 2 weeks from onset [11]. Choice between IVIG and plasma exchange is commonly shaped by feasibility and patient-specific considerations rather than strong evidence of differential efficacy; a 2023 systematic review and meta-analysis focused on severe GBS concluded that IVIG and plasma exchange have similar overall effects while IVIG is often operationally easier to deliver [12]. Combination of IVIG after plasmapheresis has not been shown to provide significant benefit in patients with GBS compared to either IVIG or plasmapheresis alone [13]. If IVIG and plasmapheresis are used in conjunction, it is vital that IVIG be administered after completion of plasmapheresis as it will remove any active IVIG thus neutralizing any potential therapeutic benefit of the IVIG treatment [2].

Escalation beyond standard therapy should be guided by evidence rather than by reflex. The guideline recommends against a second IVIG course in patients with poor prognosis and recommends against oral corticosteroids, with a weak recommendation against intravenous corticosteroids [1]. Consistent with this, the randomized, double-blind, placebo-controlled SID-GBS trial did not demonstrate benefit from a second IVIG course in poor-prognosis patients and identified a risk of serious adverse events, arguing against routine use of repeat IVIG as a strategy for early nonresponse [14]. In this case, reinitiation of IVIG was individualized, reflecting an incomplete initial course and observed neurologic improvement following plasma exchange rather than a generalized endorsement of repeat therapy. Emerging targeted therapies remain investigational; in a phase 3 randomized trial, eculizumab added to IVIG did not achieve the primary efficacy endpoint and key secondary endpoints did not reach statistical significance. This supports continued reliance on established immunotherapy and ICU-level supportive care while research seeks more effective options for the most severe phenotypes [15].

In many of the different variants of AIDP there are specific findings in the prevalence of the genders of the patients, the nerve conduction studies, and on neuroimaging. For the benefit of the readers this has been collected into a singular table (Table 2) for ease of reading and comparison and contrast of the different subtypes of AIDP.

## 4. Conclusions

Fulminant GBS should be recognized as a potentially reversible cause of apparent coma or brainstem failure. Early recognition, exclusion of central etiologies, and timely immunotherapy can result in meaningful neurologic recovery even in the most severe presentations.

## Figures and Tables

**Table 1:** Initial hospitalization course.

Timepoint	Clinical Event	Diagnostics	Intervention
Day -9	Flu-like symptoms with diarrhea	-	-
Day -2	Progressive weakness of hands and feet	-	-
Day 0 (OSH)	Initial presentation to OSH	Brain and Cervical Spine MRI, CTA Chest	Intubation and transfer
Day 0 (tertiary medical center)	Presentation to new facility after transfer, 3/5 bilateral proximal UE, 1/5 bilateral proximal LE	LP, CT Head	IVIG initiation
Day 2	Diffuse flaccid paralysis in all extremities and loss of all brainstem reflexes	CT Head, EEG	IVIG stopped, PLEX started
Day 4	-	Repeat LP	-
Day 9	Return of pupillary reflexes after 4 PLEX sessions	-	-
Day 12	Return of some SCM and trapezius activation after 5 PLEX sessions	-	-
Day 15	Minor jaw and eye movement	-	Tracheostomy placement
Day 21	PLEX completion	-	Tenth PLEX session
Day 22	Reinitiation of IVIG	-	IVIG reinitiated
Day 25	IVIG completion	-	Third day of IVIG
Day 28	Transient anisocoria and family-reported change in responsiveness, now with improved proximal extremity strength in all four limbs	CT Head	
Day 32	Patient discharged to LTAC	-	-

Abbreviations: OSH: outside hospital; MRI: magnetic resonance imagining; CT: computerized tomography; CTA: CT angiography; UE: upper extremity; LE: lower extremity; LP: lumbar puncture; IVIG: intravenous immunoglobulin; EEG: electroencephalogram; PLEX: plasma exchange; SCM: sternocleidomastoid; LTAC: long-term acute care.

**Table 2:** Overview of epidemiological and neurodiagnostic features among GBS variants.

	Global Incidence	Male:Female Prevalence	Nerve Conduction Study	Neuroimaging
Miller Fisher Syndrome	1–2/1,000,000 [1]	2:1 [16]	- Diminished sensory nerve action potentials without slowing of sensory conduction [16,17,18] - Absent H reflexes [2]	Routine imaging often has no abnormalities; however, MRI T2-weighted sequences can present with thickening and enhancement of the intrathecal nerve roots, cauda equina, and signs of central nervous system involvement [16,17,18]
Pharyngeal–Cervical–Brachial Variant	Exact incidence unknown; represents 3% of GBS cases [19]	1.3:1 [19]	- Reduced motor and possibly sensory responses with normal conduction velocities affecting the face and upper extremities more than the lower extremities [19] - Prolonged F-wave latency [20]	MRI Brain is unremarkable [20]
Acute Inflammatory Demyelinating Polyradiculoneuropathy *	1–2/100,000 [1]	1.5:1 [1]	- Slowed motor conduction velocity [21] - Conduction block or temporal dispersion [22] - Preserved compound muscle action potential amplitudes [21] - Delayed F-waves [21]	- Nerve ultrasound-enlargement of the cross-sectional area (CSA) of the nerve roots and vagus nerve can be noted, with ultrasonic sensory nerve sparing [23] - Contrast-enhanced MRI—may show enhancement of the cauda equina with preference and thickening of the ventral nerve roots; brain imaging may demonstrate enhancement of the cranial nerves, often affecting the trigeminal nerve [24]
Acute Motor Axonal Neuropathy	Exact incidence unknown; usually reported as several percentages of GBS cases due to regional variation [25]	1.5:1 [25]	- Reduced amplitudes of compound muscle action potentials without significant reduction in conduction velocity [7] - Normal sensory nerve conduction studies [21,25] - Absent F-waves [21,25]	MRI: There is frequently enhancement of the cauda equina and on T2- and T2-fat suppressed (T2FS) imaging. In addition there can be increased signal intensity of affected muscle groups [26,27]
Acute Motor-Sensory Axonal Neuropathy	Exact incidence unknown	Predominantly male; however, the exact ratio for this variant could not be identified within the literature [28]	- Reduced compound muscle action potential amplitude [29] - Reduced sensory nerve action potential amplitude [29]	MRI Brain and Cervical spine are unremarkable; however, in one case report, serial MRIs were obtained to examine a lesion in the right thalamus, which, on T2, seemed to become increasingly hyperintense as the disease progressed and then resolved when symptoms improved, hypothesized to be likely a result of an immune reaction secondary to an infectious etiology [29]

* most common form of GBS; therefore, data were reported as such when AIDP specifics were not found [19].

## Data Availability

The original contributions presented in this study are included in the article. Further inquiries can be directed to the corresponding author.

## References

[1] van Doorn P.A., Van den Bergh P.Y.K., Hadden R.D.M., Avau B., Vankrunkelsven P., Attarian S., Blomkwist-Markens P.H., Cornblath D.R., Goedee H.S., Harbo T. (2023). European Academy of Neurology/Peripheral Nerve Society Guideline on diagnosis and treatment of Guillain-Barré syndrome. Eur. J. Neurol..

[2] Shahrizaila N., Lehmann H.C., Kuwabara S. (2021). Guillain-Barré syndrome. Lancet.

[3] Leonhard S.E., Papri N., Querol L., Rinaldi S., Shahrizaila N., Jacobs B.C. (2024). Guillain-Barré syndrome. Nat. Rev. Dis. Primers.

[4] Bellanti R., Rinaldi S. (2024). Guillain-Barré syndrome: A comprehensive review. Eur. J. Neurol..

[5] Sarna M.K., Shah S., Rijhwani P., Goyal G., Jain A.K., Goel P. (2024). Guillain Barre syndrome mimicking brain death. J. R. Coll. Physicians Edinb..

[6] Bernard V., Van Pesch V., Hantson P. (2010). Guillain-Barré syndrome mimicking brain death pattern: A poorly reversible condition. Clin. Neurol. Neurosurg..

[7] Yoshimura H., Togo M., Ishii J., Ishiyama H., Tamura R., Kimura M., Kuroda T., Kusunoki S., Kawamoto M., Kohara N. (2020). Electroencephalographic findings in Bickerstaff’s brainstem encephalitis: A possible reflection of the dysfunction of the ascending reticular activating system. Clin. Neurophysiol. Pract..

[8] Kurihara T., Igarashi Y., Kobai K., Mizobuchi T., Ishii H., Matsumoto N., Yokobori S., Yokota H. (2020). Diagnosis and prediction of prognosis for Bickerstaff’s brainstem encephalitis using auditory brainstem response: A case report. Acute Med. Surg..

[9] Stoian A., Motataianu A., Bajko Z., Balasa A. (2020). Guillain-Barré and Acute Transverse Myelitis Overlap Syndrome Following Obstetric Surgery. J. Crit. Care Med..

[10] Preston D.C., Kelly J.J. (1991). “Pseudospasticity” in Guillain-Barré syndrome. Neurology.

[11] Min Y.G., Hong Y.H., Rajabally Y.A., Sung J.J. (2024). Timing of intravenous immunoglobulin treatment and outcome in Guillain–Barré syndrome: Is time nerve?. Muscle Nerve.

[12] Zaki H.A., Iftikhar H., Najam M., Masood M., Al-Marri N.D.R., Elgassim M.A.M., Fayed M., Shaban E.E. (2023). Plasma exchange versus intravenous immunoglobulin for the treatment of Guillain-Barré syndrome in patients with severe symptoms: A systematic review and meta-analysis. eNeurologicalSci.

[13] Plasma Exchange/Sandoglobulin Guillain-Barré Syndrome Trial Group (1997). Randomised trial of plasma exchange, intravenous immunoglobulin, and combined treatments in Guillain-Barré syndrome. Lancet.

[14] Walgaard C., Jacobs B.C., Lingsma H.F., Steyerberg E.W., van den Berg B., Doets A.Y., Leonhard S.E., Verboon C., Huizinga R., Drenthen J. (2021). Second intravenous immunoglobulin dose in patients with Guillain-Barré syndrome with poor prognosis (SID-GBS): A double-blind, randomized, placebo-controlled trial. Lancet Neurol..

[15] Kuwabara S., Kusunoki S., Kuwahara M., Yamano Y., Nishida Y., Ishida H., Kasuya T., Kupperman E., Lin Q., Frick G. (2024). Efficacy and safety of eculizumab in Guillain-Barré syndrome: A phase 3, multicenter, double-blind, randomized, placebo-controlled clinical trial. J. Peripher. Nerv. Syst..

[16] Rocha Cabrero F., Morrison E.H. (2023). Miller Fisher Syndrome. StatPearls.

[17] Noioso C.M., Bevilacqua L., Acerra G.M., Della Valle P., Serio M., Vinciguerra C., Piscosquito G., Toriello A., Barone P., Iovino A. (2023). Miller Fisher syndrome: An updated narrative review. Front. Neurol..

[18] Inoue N., Ichimura H., Goto S., Hashimoto Y., Ushio Y. (2004). MR imaging findings of spinal posterior column involvement in a case of Miller Fisher syndrome. AJNR Am. J. Neuroradiol..

[19] Moscona-Nissan A., López-Hernández J.C., Seidman-Sorsby A., Cruz-Zermeño M., Navalón-Calzada A. (2021). Pharyngeal-Cervical-Brachial Variant of Guillain-Barré Syndrome. Cureus.

[20] Wakerley B., Yuki N. (2013). Pharyngeal-cervical-brachial variant of Guillain-Barre syndrome. J. Neurol. Neurosurg. Psychiatry.

[21] Bourque P.R., Chardon J.W., Massie R. (2015). Autoimmune peripheral neuropathies. Clin. Chim. Acta.

[22] Marzoughi S., Marulanda L., Ngo D., Chen T. (2022). Pearls & Oy-sters: Delayed Diagnosis of Acute Motor Axonal Neuropathy With Cardiac Arrest. Neurology.

[23] Grimm A., Oertl H., Auffenberg E., Schubert V., Ruschil C., Axer H., Winter N. (2019). Differentiation Between Guillain-Barré Syndrome and Acute-Onset Chronic Inflammatory Demyelinating Polyradiculoneuritis-a Prospective Follow-up Study Using Ultrasound and Neurophysiological Measurements. Neurotherapeutics.

[24] Dodson S., Koontz N. (2019). Spinal Manifestations of Systemic Disease. Radiol. Clin. N. Am..

[25] Maqbool M., Deekshitha K., Chandana D., Abbas Z., Talukdar A., Mehdi A. (2024). Postpartum Guillain-Barré Syndrome Presenting as Acute Motor Axonal Neuropathy in a Young Female: A Report of a Rare Case. Cureus.

[26] Phillips J., Kincaid J., Garg B. (1997). Acute Motor Axonal Neuropathy in Childhood: Clinical and MRI findings. Pediatr. Neurol..

[27] Berciano J., Gallardo E., Fernández-Torre J.L., González-Quintanilla V., Infante J. (2011). Magnetic resonance imaging of lower limb musculature in acute motor axonal neuropathy. J. Neurol..

[28] Zafar A., Ali S., Gazi Z.U., Iqbal A., Khan S., Shoaib M. (2022). Classification of Guillain-Barré syndrome based on electrophysiological features. Int. J. Health Sci..

[29] Geng N., Wang P., Zhang Y. (2023). Acute Motor-Sensory Axonal Polyneuropathy Variant of Guillain-Barré Syndrome with a Thalamic Lesion and COVID-19: A Case Report and Discussion on Mechanism. Front. Neurol..

